# Patterns of weariness-related symptoms in Parkinson’s disease: impact of disease progression and levodopa treatment

**DOI:** 10.1016/j.prdoa.2026.100442

**Published:** 2026-04-11

**Authors:** Danielle Hersonsky, Assaf Benesh, Edmund Ben-Ami, Eitan Raveh

**Affiliations:** a The faculty of Medical and Health Science, Tel-Aviv University, Tel Aviv, Israel; b School of Computer Science and Artificial Intelligence, Tel Aviv University, Israel; c NeuraLight LTD, Tel Aviv, Israel

**Keywords:** Non-motor symptoms, Dopaminergic treatment, Sleep disorders, Quality of life

## Abstract

**Background:**

Weariness-related symptoms (WRS), i.e. fatigue, daytime sleepiness, and sleep problems are common non-motor symptoms in Parkinson’s disease (PD) that impair quality of life. Although related, they represent distinct clinical domains, and the role of dopaminergic therapy in their progression remains unclear. We compared stable levodopa regimens with levodopa dose escalation to evaluate their impact on WRS evolution.

**Methods:**

From a database of 1,521 PD patients, 159 individuals (mean age 64.7 ± 9.36 years; 61.7% male; Hoehn and Yahr stage 0–2) with two assessments 6–18 months apart were included. Patients were grouped into stable medication regimen (SMR) or increased levodopa dose (ILD). Longitudinal changes in fatigue, daytime sleepiness, and nighttime sleep problems were analyzed, including age-stratified comparisons.

**Results:**

In the SMR group, daytime sleepiness increased significantly over time (0.21 ± 0.86, P = 0.001), while fatigue and sleep problems remained stable. In contrast, the ILD group showed a significant increase in fatigue (0.15 ± 0.89, P = 0.017), with no changes in daytime sleepiness or sleep problems. Worsening daytime sleepiness under stable medication was confined to older patients (>65 years, P = 0.003), whereas fatigue worsening after levodopa escalation was mainly observed in younger patients (≤65 years, P = 0.03).

**Conclusion:**

Daytime sleepiness appears disease-driven and independent of medication changes, while fatigue is more closely associated with levodopa escalation, supporting individualized management and targeted therapeutic development.

## 1 Introduction

Parkinson's disease (PD) is a complex neurodegenerative disorder primarily characterized by heterogeneous motor symptoms, including tremor, rigidity, bradykinesia and postural instability and gait impairment [1]. Patients with PD also commonly experience a wide range of non-motor symptoms [2], [3], which can significantly impact their quality of life [2], [4], [5], [6], [7]. Among these symptoms, weariness-related symptoms (WRS) are widely reported. These include ‘sleep problems’, having trouble going to sleep at night or staying asleep through the night, ‘daytime sleepiness’, having trouble staying awake during the daytime, and the most known one – fatigue. [7], [8], [9], [10], [11], [12], [13], [14]. It is important to acknowledge that fatigue in PD is described as a lack of energy or a need for increased effort in order to attempt daily activities that is distinct from sleepiness, lack of motivation, and depression [15]. Nevertheless, these symptoms display complex patterns across the disease course, as they are known to be influenced by both disease progression [16], [17] and dopaminergic treatment [14], [18]. Furthermore, each of the aforementioned symptoms presents a different pattern compared with the others, e.g. fatigue severity was reported to worsen with disease progression [19], [20], while sleep problems are reported to worsen as levodopa intake increases [18]. As a result, the sactive effects of disease progression and dopaminergic treatment on each of those WRS are often mixed and misunderstood [7], [17], [21]. Therefore, the objective of our study was to investigate the effect of dopaminergic treatment and PD progression on sleep problems, daytime sleepiness and fatigue among patients with PD. Considering the significant impact of WRS on patients' quality of life, gaining a clearer understanding of how these factors affect these symptoms could add an important dimension to our understanding of this devastating disease.

## 2 Methods

We used data from the Parkinson Progression Marker Initiative (PPMI), which is widely used for retrospective analysis of PD progression[22], [23]. Inclusion criteria were medical records of PPMI participants diagnosed with PD, who were classified as Hoehn and Yahr (H&Y) [24] stage ≤ 2, and had an interval of 6–18 months between consecutive assessments of the Movement Disorder Society Unified Parkinson’s Disease Rating Scale (MDS-UPDRS) Part I [25]. We excluded individuals receiving medications known to affect WRS, including sedative–hypnotics (e.g., zolpidem, melatonin), benzodiazepines (e.g., clonazepam, lorazepam, alprazolam), antidepressants (SSRIs and others; e.g., fluoxetine, sertraline, paroxetine, escitalopram, trazodone, mirtazapine), antihistamines (cetirizine), opioid analgesics (tramadol), anticonvulsants (gabapentin), dopaminergic agents (carbidopa, amantadine), α1-blockers (tamsulosin), 5α-reductase inhibitors (finasteride), calcium channel blockers (amlodipine), and β-blockers (e.g., metoprolol, propranolol, bisoprolol, atenolol, carvedilol) [26]. As a part of the MDS-UPDRS I assessment, we analyzed the specific WRS e.g. sleep problems (NP1SLPN; item 1.7), daytime sleepiness (NP1SLPD; item 1.8) and fatigue (NP1FATG; item 1.13), as self-reported by the participants. All these items are scored on a 5-point scale ranging from 0 (“normal”) to 4 (“severe”). For each case, we analyzed a series of consecutive visits, in 2 different scenarios: Visits where no change in medications regimen occurred over time (Stable Medication Regimen, SMR group), and visits with an increase in Levodopa dosage (and not other dopaminergic medications) between consecutive assessments (Increased Levodopa Dosage, ILD group). Regarding concomitant medications, for both SMR and ILD groups visits in which the participants took any medication that was documented as having a sedative effect were excluded. A list of concomitant medications is provided in Appendix ISupplementary Material (Supplementary Material). The groups were defined according to change in the Levodopa Equivalent Daily Dose (LEDD) measure [27], with all other PD medications unchanged. To analyze the change in WRS in each of these groups, we randomly chose adjacent visits with a predefined time interval (175–190 days between visits) from each group, and calculated the difference in WRS using a one-sample *t*-test to determine whether mean item differences between visits were significantly greater than zero. The time interval of 175–190 days (∼half a year) was chosen since it was sufficiently long to capture a measurable effect of medication changes, while also reflecting the longitudinal effect of the PD progression itself. Shorter intervals may be insufficient to detect meaningful symptom changes, whereas longer intervals risk confounding by disease progression to a greater degree. To ensure non-overlapping and independent observation windows we implemented the visits pairs choosing in the following way: after filtering all the adjacent visit pairs that were eligible, picked only the first group of disjointed ones (for example, if visits (1,2), (2,3), (3,4), (5,6) were eligible, we kept (1,2), (3,4), (5,6)). Then we chose the first valid group of the available. In addition, since WRS in Parkinson's disease are influenced by numerous potential confounders, including age, disease duration, medications, disease severity and more, we performed a multivariable analysis. The covariates we chose were: levodopa group (SMR vs ILD, coded 0 and 1 respectively), time since diagnosis, LEDD value in the visit, Hoehn & Yahr score, age group (age<=65 or age > 65, coded 0 and 1 respectively) and visits order (whether the SMR visits were before the ILD once or the other way around). Our outcome variables in each model were the within-patient change in item score between the two visits of each medication condition (SMR and ILD). A random intercept was included for each patient to account for the repeated-measures structure of the data. Age group was assigned based on the patient's age at their earliest available visit. All models were estimated using restricted maximum likelihood.

To address the risk of false-positive findings arising from multiple comparisons, P-values from all hypothesis tests were adjusted using the Benjamini-Hochberg false discovery rate procedure. Statistical significance was defined as a mean difference greater than zero with *P* < 0.05.

## 3 Results

Out of a total of 1,521 patients, 159 had both SMR and ILD visits pairs in the required time intervals, and the rest (1,362 patients) were excluded. The patients’ demographic and clinical characteristics are presented in Table 1.

**Table 1:** Demographic and clinical characteristics of the study population.

	**Study Population: N = 159**			
Males	110 (69.2%)			
Females	49(30.8%)			

	**SMR Group**		**ILD Group**	
	**Visit 0**	**Visit 1**	**Visit 0**	**Visit 1**
Age (years)	65.54 ± 9.58	66.04 ± 9.58	65.22 ± 9.16	65.71 ± 9.16
Time since diagnosis (months)	3.74 ± 3.32	4.238 ± 3.32	3.42 ± 2.55	3.92 ± 2.55
MDS-UPDRS I (mean ± SD)	7.65 ± 5.27	8.23 ± 5.26	7.71 ± 4.47	7.74 ± 4.97
MDS-UPDRS II (mean ± SD)	8.39 ± 5.84	9.20 ± 6.24	8.75 ± 5.13	8.94 ± 5.56
MDS-UPDRS III (mean ± SD)	21.01 ± 11.06	23.60 ± 11.34	25.86 ± 10.30	23.64 ± 10.40
LEDD (mean ± SD)	419.75 ± 388.39		320.71 ± 391.99	572.89 ± 401.15
ΔLEDD (mean ± SD)	0		252.19 ± 198.04	
H&Y stage	1.79 ± 0.42	1.79 ± 0.44	1.86 ± 0.35	1.84 ± 0.38
Stage 2 N(%)	127 (79.9%)	128 (80.5%)	137 (86.2%)	135 (84.9%)
Stage 1 N(%)	31 (19.5%)	29 (18.2%)	22 (13.8%)	23 (14.5%)
Stage 0 N(%)	1 (0.6%)	2 (1.26%)	0	1 (0.6%)

H&Y:Hoehn and Yahr; ILD:Increased Levodopa Dosage; LEDD:Levodopa Equivalent Daily Dose; MDS-UPDRS:Movement Disorder Society–Unified Parkinson's Disease Rating Scale; SD:Standard Deviation; SMR:Stable medication regimen.

**Fig. 1:**
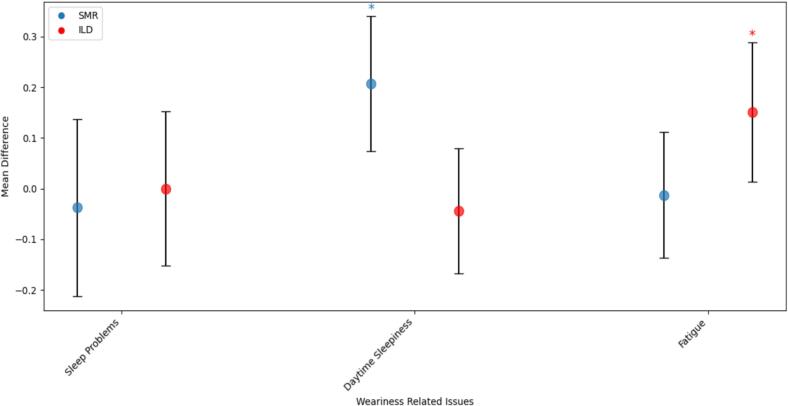
Changes in weariness-related symptoms in patients with stable medication regimens (SMR, blue) or Increased Levodopa Dosage (ILD, red). Each point depicts the mean yearly progression and 95% confidence interval. Significance is denoted by asterisks if P < 0.05.

When analyzing the patients according to potential change in medications over time, daytime sleepiness significantly increased over time for patients with stable medication regimens (SMR group, 0.21 ± 0.86, P = 0.0013), whereas no significant changes were detected for patients with an increase in levodopa dosage (ILD group, 0.00 ± 0.98, P = 0.50). Fatigue increased significantly in the ILD group (0.15 ± 0.89, P = 0.0167), however no significant changes were observed in the SMR group **(**−0.01 ± 0.80, P = 0.579). As for change in sleep problems, no significant changes were found in both the SMR and the ILD groups **(**−0.04 ± 1.12, P = 0.663; −0.04 ± 0.79, P = 0.758, respectively). Changes in WRS are depicted in Fig. 1.

When stratifying the data according to the patients’ age in the first visit (Younger-age ≤ 65, older-age > 65), younger patients showed a significant increase in fatigue under increased levodopa comparison (0.27 ± 0.91, P = 0.047), while when comparing the differences for sleep problems and daytime sleepiness between visits with increased levodopa, no significant change was observed (−0.17 ± 1.00, P = 0.958 for sleep problems; −0.11 ± 0.75, P = 0.958 for daytime sleepiness). As for the comparison between visits with same medications for younger patients, no significant change was documented for any of the symptoms (0.04 ± 0.80, P = 0.564 for sleep problems; 0.15 ± 0.84, P = 0.249 for daytime sleepiness; −0.13 ± 0.61, P = 0.958 for fatigue).

**Fig. 2:**
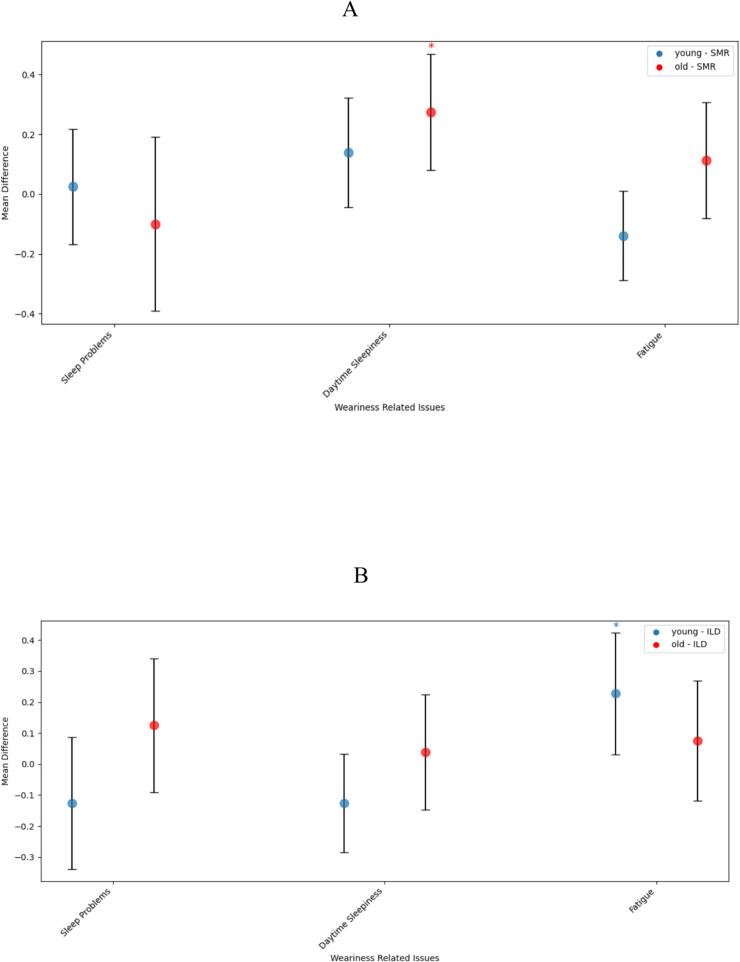
Changes in weariness-related symptoms in patients stratified according to age (Younger in blue, older in red) with either stable medication regimens (SMR, Image A) or Increased Levodopa Dosage (ILD, Image B). Each point depicts the mean yearly progression and 95% confidence interval. Significance is denoted by asterisks if P < 0.05.

When observing the older group, under no medication change, a significant increase in daytime sleepiness was seen (0.25 ± 0.87, P = 0.047), while no significant change was observed in the other symptoms (−0.10 ± 1.33, P = 0.958 for sleep problems; 0.08 ± 0.91, P = 0.499 for fatigue). When comparing changes in the WRS between visits in which levodopa was increased, no significant change was observed for any of the symptoms (0.14 ± 0.95, P = 0.272 for sleep problems; 0.01 ± 0.82, P = 0.673 for daytime sleepiness; 0.06 ± 0.86, P = 0.538 for fatigue). Results are depicted in Fig. 1, Fig. 2.

Results of the Multivariable linear mixed-effects model of weariness-related MDS-UPDRS Part I items are presented in Table 2.

**Table 2:** Multivariable Linear Mixed-Effects Model Results for Weariness-Related MDS-UPDRS Part I Items.

	Sleep Problems		Daytime Sleepiness		Fatigue	
	β	P	β	P	β	P
Intercept	0.41	0.009a	0.48	0.00 a	0.15	0.215a
H&Y centered	0.046	0.74	0.0095	0.93	−0.091	0.41
Age group	−0.10	0.49	0.17	0.16	0.28	0.017
ΔLEDD	−0.0006	0.10	0.0003	0.29	−0.0003	0.35
DA non-ergoline taken	−0.16	0.22	0.12	0.28	−0.087	0.42
Group order	0.056	0.60	−0.076	0.39	−0.16	0.063
Levodopa group	0.013	0.94a	−0.31	0.043a	0.46	0.005a
Time since diagnosis	0.045	0.019	0.021	0.18	0.035	0.025
Age X levodopa interaction	0.321	0.181a	0.122	0.510	−0.388	0.043a

^a^FDR-adjusted p-value (Benjamini-Hochberg correction across 9 primary hypothesis tests). All other p-values are uncorrected and represent control variables rather than primary hypotheses. H&Y:Hoehn and Yahr stage; LEDD:levodopa equivalent daily dose; DA:dopamine agonist; MDS-UPDRS:Movement Disorder Society–Unified Parkinson's Disease Rating Scale.

Significant findings remained consistent after controlling for covariates (Table 2); The SMR group has a significant increase in daytime sleepiness (β = 0.477, 95% CI [0.253, 0.700], p < 0.001), and the ILD group has a significant increase in fatigue score (β = 0.463, 95% CI [0.187, 0.740], p = 0.005). Interestingly, the ILD group has a significant negative effect on daytime sleepiness (levodopa was associated with less daytime sleepiness; β = -0.311, 95% CI [-0.581, −0.041], p = 0.043). These remained significant after applying Benjamini Hochberg correction. Moreover, following our stratification analysis, there’s a significant interaction between the age groups and fatigue (β = -0.388, 95% CI [-0.718, −0.059], p = 0.043) − the effect seems to be driven by the younger group (for age ≤ 65 (age_group = 0) β = 0.463), whereas for age > 65 (age_group = 1): β = 0.075. Among the covariates, age group was significantly associated with fatigue change (β = 0.288, p = 0.017), as older patients show greater fatigue increases independent of levodopa group. Additionally, time since diagnosis was significantly associated with sleep problems (β = 0.045, p = 0.019) and fatigue (β = 0.035, p = 0.025), suggesting that disease duration independently contributes to weariness-related symptom progression.

## 4 Discussion

Weariness-related symptoms represent some of the most prevalent and disabling non-motor features of PD [20], [21], [28]. Despite extensive investigation over recent decades, there is still no consensus regarding the relative contributions of disease progression and dopaminergic treatment to the evolution of these symptoms [7]. Previous studies have variably linked WRS to neurodegeneration, medication exposure, aging, and comorbid non-motor burden [12], [29], [30], [31] underscoring their heterogeneity in PD. Being the most prominent symptom among WRS, fatigue represents this heterogenic nature in previous literature [19], [20], [32], [33]. While some studies attribute the worsening of fatigue primarily to PD progression and report symptomatic improvement with dopamine agonists, others suggest that fatigue may be driven mainly by the direct effects of dopaminergic therapy and therefore question the contribution of disease-related mechanisms. For example, in a prospective follow-up of 9 years, Ongre and colleagues have found that 191 PD patients had significantly more fatigue than healthy controls from baseline, though a large proportion of patients had a significant increase or decrease in fatigue score between consecutive visits [16]. The researchers also found that lower levels of fatigue were associated with the use of dopamine agonists. In our study, fatigue seemed to be more affected by dopaminergic treatment than by disease progression. This trend aligns with the previous work of Dobryakova and colleagues, who reviewed multiple structural and functional neuroimaging studies, suggesting that fatigue is most affected by imbalance of dopamine rather than a direct progress of the disease [33]. This distinction is important because fatigue, while highly prevalent and strongly associated with reduced quality of life in PD, remains one of its least characterized non-motor symptoms [20]. Clarifying the roles of endogenous dopaminergic dysfunction and dopaminergic therapy in the development and progression of fatigue has the potential to substantially inform therapeutic decision-making and improve patient quality of life.

Another important symptom described in PD is daytime sleepiness. While many papers focus on the induction of daytime sleepiness due to dopaminergic treatment [34], [35], others emphasize the effect of the disease itself. In their 8-year follow-up study [36], Gjerstad and colleagues showed that the prevalence of daytime sleepiness increased from 5.6% at baseline to over 50%, concluding that both age and disease related disturbances of the sleep-wake regulation contribute to hypersomnia in PD. Our findings partially align with these results, as in our study, daytime sleepiness seems to be mostly affected by disease progression, emphasizing the importance of future exploration of different neurological pathways on the symptom. Particularly, we did not examine the effect of dopamine agonists specifically, which may represent an additional contributing pathway warranting further investigation. It is important to mention that this pattern is consistent with progressive degeneration of wake-promoting neuronal systems within the brainstem and hypothalamus [21], including both dopaminergic and non-dopaminergic pathways. Structural and functional imaging studies further implicate regions such as the anterior insula and orbitofrontal cortex in the subjective experience of sleepiness, linking neurodegeneration directly to its progression [37]. The observed association between daytime sleepiness and disease duration and severity supports the interpretation that daytime sleepiness reflects intrinsic disease mechanisms rather than a secondary consequence of medication or nighttime sleep disruption [35], [38], [39].

The absence of significant progression of sleep problems in our cohort may be unexpected, given the high prevalence of insomnia and fragmented sleep in PD [40], [41]. This may also reflect the relatively early disease stage of the PPMI participants (H&Y ≤ 2), in whom sleep disturbances may not yet show pronounced longitudinal worsening over short follow-up periods. However, prior longitudinal studies indicate that not all weariness-related symptoms progress uniformly. While the number of coexisting sleep disturbances may increase with disease duration [27], [33], [42], individual sleep complaints, and particularly insomnia, often exhibit fluctuating or plateauing courses over shorter follow-up periods [20], [32]. In contrast, fatigue and daytime sleepiness appear to evolve more dynamically [43], reinforcing the notion that these domains may provide more sensitive markers of short-term disease progression or treatment effects.

Stratification by age provided further insight into the relative contributions of disease progression and medication effects. Among older patients, the progression of daytime sleepiness in the stable-medication group remained significant, reinforcing the notion that daytime sleepiness is a disease-driven phenomenon that is amplified by aging. This finding is consistent with evidence that older age and longer disease duration are among the strongest predictors of excessive daytime sleepiness in PD. In contrast, among younger patients, fatigue worsened significantly only following levodopa dose escalation. Because younger individuals typically have fewer age-related contributors to sleep-wake dysregulation and shorter disease duration, this pattern likely isolates the effect of dopaminergic treatment on fatigue more clearly [44]. This does not mean older people’s fatigue is not affected by increased levodopa treatment, as the non-significant p-value may simply reflect insufficient statistical power rather than a true medical effect.

The multivariate linear mixed-effects analysis confirmed that the primary findings were robust to confounding. After controlling for disease duration, Hoehn & Yahr stage, LEDD change, dopamine agonist use, and visit order, levodopa dose escalation remained independently associated with increased fatigue, and the stable medication group showed a significant independent increase in daytime sleepiness. Interestingly, the multi-variate analysis revealed a significant negative association between levodopa dosage increase and daytime sleepiness, which may be explained by better motor control that may lead to better sleep quality overnight, possibly reducing the daytime sleepiness [45]. It may be counterintuitive, considering the significant increase in fatigue, though it makes sense since fatigue and daytime sleepiness are not necessarily correlated/affecting each other. The significant negative association between levodopa dose escalation and daytime sleepiness further suggests that these two weariness-related symptoms are differentially affected by dopaminergic treatment.

The observed differences between weariness-related domains − where some symptoms appear to track disease progression while others are more closely affected by dopaminergic therapy − implies that the construct commonly referred to as “fatigue” in PD represents a substantially more complex phenomenon. Rather than reflecting a singular dimension of physical or mental tiredness, or merely a side effect of dopaminergic exposure, fatigue likely encompasses overlapping components of reduced arousal and sedation along with daytime sleepiness and sleep problems. The relative stability of sleep problems suggests that daytime sleepiness and fatigue may serve as more sensitive outcome measures in short-term longitudinal studies.

These findings correspond with the observations regarding L-Dopa-induced sedation, which highlighted early on that dopaminergic treatment can modulate alertness independently of motor benefit [45], [46]. Together, these results support the notion that weariness-related symptoms in PD arise from partially distinct and interacting mechanisms. Future longitudinal and interventional studies will therefore require more refined and component-specific assessment of weariness-related symptoms in order to more accurately isolate disease progression and treatment effects.

A key strength of this study is the longitudinal assessment of multiple weariness-related non-motor symptoms in a well-characterized PD cohort with detailed medication data. Several limitations should nonetheless be acknowledged. First, residual confounding is possible, as levodopa dose escalation may reflect underlying disease or non-motor burden rather than a direct treatment effect. Second, weariness outcomes were based on patient-reported MDS-UPDRS items, which are subjective and may not capture the full spectrum of non-motor or weariness-related changes; in particular, nighttime sleep problems were not assessed using objective measures such as polysomnography or actigraphy. Third, the relatively short follow-up interval may have limited detection of longer-term symptom trajectories. Finally, the observational design precludes causal inference, and the underlying mechanisms linking disease progression, dopaminergic treatment, and weariness-related symptoms remain to be clarified. In addition, regarding the age stratification, and due to the smaller subgroup sample sizes, it is important to acknowledge that a non-significant p-value may reflect insufficient statistical power rather than a true absence of effect. The observed difference in significance between age groups may relate to a quantitative difference in effect magnitude, or simply reduced power, rather than a qualitative distinction in the underlying mechanism. Larger studies specifically powered for age-stratified analyses are needed to clarify whether the association between levodopa dose escalation and fatigue genuinely differs between younger and older patients with PD. Finally, we did not analyze the presence of sleep related disorders such as REM sleep disorder or sleep apnea, which are relatively common in the general population (particularly among PD patients), and could independently contribute to WRS or interact with dopaminergic treatment effects.

## 5 Conclusion

Our findings suggest that WRS are heterogeneous with respect to their underlying mechanisms. Specifically, daytime sleepiness in PD may reflect disease progression independent of medication changes, whereas fatigue appears more closely associated with levodopa dose escalation, potentially indicating treatment-related side effects or increasing disease burden. In contrast, nighttime sleep quality remained stable over the study period. Recognizing these dissociable symptom trajectories has important implications for clinical monitoring, trial design, and mechanistic research into non-motor symptoms of PD. These results highlight the need for reliable tools capable of measuring WRS in a manner that captures their distinct underlying mechanisms. Accurate assessment of disease-related weariness may support more individualized treatment dosing, enable longitudinal monitoring of a symptom domain that is highly burdensome for patients, and facilitate the development of targeted therapeutic interventions. Future studies should aim to disentangle disease-driven and treatment-related mechanisms through longer follow-up periods, incorporation of objective sleep and arousal measures, and more refined phenotyping of fatigue and sleepiness domains, and should also assess the effects of other PD medications, including dopamine agonists and COMT/MAO-B inhibitors.

## 6 Funding sources and conflict of interest

No specific funding was received for this work. DH declares no conflict of interests. ER, AB and EBA are all employees of NeuraLight.

Financial disclosures for the previous 12 months.

The authors declare that there are no additional disclosures to report.

## 7 Ethical compliance statement

Data used in the preparation of this article was obtained on 2025–01-10 from the Parkinson’s Progression Markers Initiative (PPMI) database (https://www.ppmi-info.org/access-dataspecimens/download-data), RRID:SCR_006431. Parkinson’s Progression Markers Initiative (PPMI) is supported by The Michael J. Fox Foundation for Parkinson’s Research and Aligning Science Across Parkinson’s (ASAP), with additional funding from AcureX, Allergan, Amathus Therapeutics, Avid Radiopharmaceuticals, AskBio, BIAL, BioArctic, Biohaven, BioLegend, Biogen, BlueRock Therapeutics, Bristol Myers Squibb, Calico Labs, Capsida Biotherapeutics, Celgene, Cerevel Therapeutics, Coave Therapeutics, DaCapo Brainscience, Denali, the Edmond J. Safra Foundation, Eli Lilly, Gain Therapeutics, GE Healthcare, Genentech, Golub Capital, GSK, Handl Therapeutics, Insitro, Jazz Pharmaceuticals, Johnson & Johnson Innovative Medicine, Lundbeck, Meso Scale Discovery, Merck, Mission Therapeutics, Neurocrine Biosciences, Neuron23, Neuropore, Pfizer, Piramal, Prevail Therapeutics, Roche, Sanofi, Servier, Sun Pharma Advanced Research Company, Takeda, Teva, UCB, Vanqua Bio, Verily, Voyager Therapeutics, The Weston Family Foundation, and Yumanity Therapeutics.

## CRediT authorship contribution statement

**Danielle Hersonsky:** Writing – original draft, Formal analysis, Conceptualization. **Assaf Benesh:** Writing – review & editing, Formal analysis, Conceptualization. **Edmund Ben-Ami:** Writing – review & editing, Formal analysis, Conceptualization. **Eitan Raveh:** Writing – review & editing, Supervision.

## Declaration of competing interest

The authors declare the following financial interests/personal relationships which may be considered as potential competing interests: [Danielle Hersonsky Sarid declares no conflict of interests. Assaf Benesh, Edmund Ben-Ami and Eitan Raveh are all employees of NeuraLight].
