# Supplementary material for: Patterns of weariness-related symptoms in Parkinson’s disease: impact of disease progression and levodopa treatment
Source: Clin Park Relat Disord. 2026 Apr 11;14:100442. doi: 10.1016/j.prdoa.2026.100442 (PMC13092868; doi:10.1016/j.prdoa.2026.100442)
Supplement: Supplementary Data 1 [file mmc1.docx]

Appendix I: List of concomitant medications

**Parkinson’s disease medications**: Levodopa, MAO Inhibitor, DA Non-ergoline, DA Ergoline, COMT Inhibitor

**General Concomitant Medications**: Acetaminophen, Acyclovir, Albuterol, Alendronate, Alfuzosin, Allopurinol, Alprazolam, Amantadine, Amitriptyline, Amlodipine, Amoxicillin, Apixaban, Atenolol, Atorvastatin, Benztropine mesylate, Biperiden hydrochloride, Bisoprolol, Botulinum toxin, Brimonidine, Bupropion, Candesartan, Carbidopa, Carvedilol, Celecoxib, Cetirizine, Ciprofloxacin, Citalopram, Clonazepam, Clopidogrel, Colchicine, Cyclosporine ophthalmic, Diazepam, Diclofenac, Digoxin, Diltiazem, Donepezil, Dorzolamide-timolol, Doxazosin, Doxepin, Duloxetine, Empagliflozin, Enalapril, Eplerenone, Escitalopram, Esomeprazole, Estradiol, Ezetimibe, Famotidine, Felodipine, Fenofibrate, Ferrous sulfate, Fesoterodine, Fexofenadine, Finasteride, Fluocinonide, Fluoxetine, Fluticasone, Furosemide, Gabapentin, Glimepiride, Glycopyrronium, Hydrochlorothiazide, Hydrocortisone cream, Ibandronate, Ibuprofen, Irbesartan, Ketoconazole, Lansoprazole, Latanoprost, Lenalidomide, Levetiracetam, Levothyroxine, Lisinopril, Lithium, Loperamide, Loratadine, Lorazepam, Losartan, Lovastatin, Meclizine, Meloxicam, Memantine, Mesalamine, Metformin, Methotrexate, Methylphenidate, Metoprolol, Metronidazole, Midodrine, Mirabegron, Mirtazapine, Modafinil, Mometasone, Montelukast, Morphine, Naproxen, Nitrofurantoin, Omeprazole, Ondansetron, Oxybutynin, Pantoprazole, Paroxetine, Phenprocoumon, Pravastatin, Prednisone, Pregabalin, Propranolol, Quetiapine, Quinapril, Quinine, Ramipril, Ranitidine, Rivastigmine, Rosuvastatin, Rytary, Salmeterol, Scopolamine, Sertraline, Sildenafil, Simvastatin, Solifenacin, Spironolactone, Tadalafil, Tamsulosin, Terbinafine, Testosterone, Timolol, Tolterodine, Tramadol, Trazodone, Triamterene, Trospium, Valacyclovir, Valsartan, Venlafaxine, Verapamil, Warfarin, Zaleplon, Zolpidem
